# Behavioral, Emotional and School Adjustment in Adolescents with and without Developmental Language Disorder (DLD) Is Related to Family Involvement

**DOI:** 10.3390/ijerph17061949

**Published:** 2020-03-16

**Authors:** Mario Valera-Pozo, Daniel Adrover-Roig, Josep A. Pérez-Castelló, Victor A. Sanchez-Azanza, Eva Aguilar-Mediavilla

**Affiliations:** Universitat de les Illes Balears-Cta. Valldemossa, Km. 7.5-07122 Palma, Spain; m.valera@uib.es (M.V.-P.); daniel.adrover@uib.es (D.A.-R.); pep.perez@uib.es (J.A.P.-C.); v.sanchez@uib.es (V.A.S.-A.)

**Keywords:** language delay, specific language impairment, family involvement, mental health, school adaptation

## Abstract

Developmental language disorder (DLD) refers to a language delay in the absence of other underlying causes. Individuals with DLD can also present other problems related to behavioral, scholarly, and emotional aspects of their daily lives because of their language difficulties. Moreover, these difficulties could be influenced by family and socioeconomic characteristics. Twenty-eight bilingual adolescents with and without DLD in typical schools were followed from childhood to adolescence. At age five, language and cognitive variables were assessed. In addition, language, behavior, emotional and school adjustment, and socioeconomic and family aspects were evaluated at age 12. Results reveal that adolescents with DLD show poorer school adjustment and less adaptive skills when evaluated by their tutors, and a larger index of emotional problems when self-assessed. Moreover, family involvement, but not socioeconomic status (SES), emerged as a protective factor since it was related to behavioral, emotional, and school adjustment, a result that was further confirmed by structural equation modeling. Therefore, a more global approach involving individuals, schools and families is needed to provide adolescents with DLD adequate support. It is important to stimulate their social skills and emotional adjustment so they can cope with social difficulties more easily, especially at school.

## 1. Introduction

Persons with developmental language disorder (DLD; [1,2]), previously named specific language impairment (SLI; [3]), show a language delay despite the fact that other aspects that underlie language difficulties are within the normal rates. Thus, the DLD diagnostic criterion is defined as a persistent language delay not resolved at age five, which affects everyday life communication and/or learning, without the presence of a medical condition, such as brain injury, genetic conditions or chromosome disorders, hearing loss, autism spectrum disorders or intellectual disability. Not only does DLD affect a person’s everyday life communication, but also many aspects related to it, such as social skills, leadership or adaptive skills that finally could affect their quality of life [4,5,6,7]. For instance, individuals with DLD display worse performance on different social and adaptive situations and they use more inappropriate conflict resolution strategies compared to typically developing persons [8]. As a result, children and adolescents with DLD show several adjustment problems, including behavioral and emotional difficulties, in comparison with their peers [9,10,11,12,13,14].

Referring to pre-school children with DLD, previous results show more externalization difficulties such as hyperactivity, aggressiveness and even delinquency [15], as well as attention problems when performing auditory tasks [16,17]. With respect to children and adolescents with DLD, they have been shown to be twice as likely to show internalizing difficulties, and more than twice as likely to show externalizing difficulties [11] as compared to typical developing individuals. Moreover, early language difficulties are known to be a good predictor of emotional problems in late childhood and adolescence [16]. In this vein, and since emotional problems seem to increase from childhood to adolescence, anxious and depressive traits are common among teenagers with this disorder [16,18]. Therefore, adolescents with DLD are more vulnerable to emotional disturbances than their typical peers [19] and report higher indexes of internalized and externalized emotional difficulties, more (and more severe) emotional symptoms, and poorer psychological functioning than typical children and adolescents [11,20,21]. In some cases, those behavioral and emotional symptoms reach clinical ranks and need specific attention. For example, young adults with language difficulties have thrice more possibilities of presenting social anxiety (previously called social phobia) than typical peers [22]. Furthermore, experiencing problems in emotional regulation and difficulties in communicating about one’s emotions and the emotions of others are some of the factors that seem to underlie emotional difficulties [18,23,24]. In this sense, a curious phenomenon occurs in persons with DLD: while they inform similar levels of social skills than their peers in self-reports, they suffer more stress than the others in social situations [25]. This might be explained because of their language difficulties and perhaps lower self-esteem, especially when children with DLD grow up. Moreover, previous results show that pre-adolescents and adolescents with DLD, but not children, report lower self-esteem indexes than typical adolescents, and they even perceive themselves as shyer than others [26,27]. However, these lower self-esteem scores tend to disappear when they finish compulsory education, matching that of their normative peers [27]. Taken together, these studies highlight that not all individuals with DLD experience the same difficulties in behavioral, emotional and social adjustment, and the persistence of the aforementioned difficulties is inconsistent over time [28]. Therefore, exploring how language difficulties, confirmed at age five, influence later scholar and adaptative development during a stage that has not been paid sufficient attention in DLD (beginning of adolescence) is relevant in the context of this disorder. 

In this sense, previous studies have shown that family variables could be related to both language difficulties and personal adjustment [20,28]. The socioeconomic status (SES) of the family and their own family involvement are variables of particular interest in cases of language deficits. Regarding the former, the interrelation of factors such as the academic level attained and the economic position (occupation and income) of the family, that is, SES, is related up to a certain degree with language development and language delay [29,30,31,32]. For instance, the Catalise consortium include social difficulties (poverty and low level of parental education) as a risk factor of a developmental language disorder [1,2]. Other authors [33] state that those children from low SES families are exposed to much fewer words than those from high SES families. For that reason, their vocabulary acquisition could be compromised and their language development could be affected compared to their peers. Thus, several authors have argued that oral language development has become an important variable to explain the relationship between SES and elementary school performance [34]. Moreover, DLD is a disorder that has been found more frequently in individuals growing up in a low sociocultural context [29,35]. For that reason, SES and language disorders have a relationship that must be observed carefully.

Family involvement is another phenomenon that requires further study in DLD. Although conceptualizing family involvement is difficult [36], we can define it as the group of parental behaviors, concerns and participation in both school and home in order to help children in their educational process [37]. Aguado [30] points out that rejection or the occurrence of negligence are important factors that might explain associated difficulties to DLD in families with low involvement. In this sense, previous studies have found more insecure patterns of attachment in children with DLD than in their peers [38]. Related to this, different studies [31,32] have shown that low stimulant contexts or contexts with low-quality language inputs are relevant factors to the appearance of language difficulties. In fact, rejection, lack of affection and negligence during education could be considered as forms of maltreatment which might drive to poorer language development [39]. Alternatively, family involvement can also have a positive influence on several aspects of children’s well-being. As such, family involvement has shown a positive relationship with self-esteem [40], an important protective factor against most diseases. Moreover, high family involvement is negatively related to behavioral problems and positively associated with social skills, and children with larger family involvement display fewer behavioral problems and better social and school adjustment [37,41]. Referring to language, children in highly involved families are exposed to more and richer language input, which might turn into a protective factor to prevent the development of different language disorders in the future [42]. For these reasons, family involvement could be a very important protective factor in DLD, not only under a linguistic point of view but also in terms of mental health.

**Figure 1:**
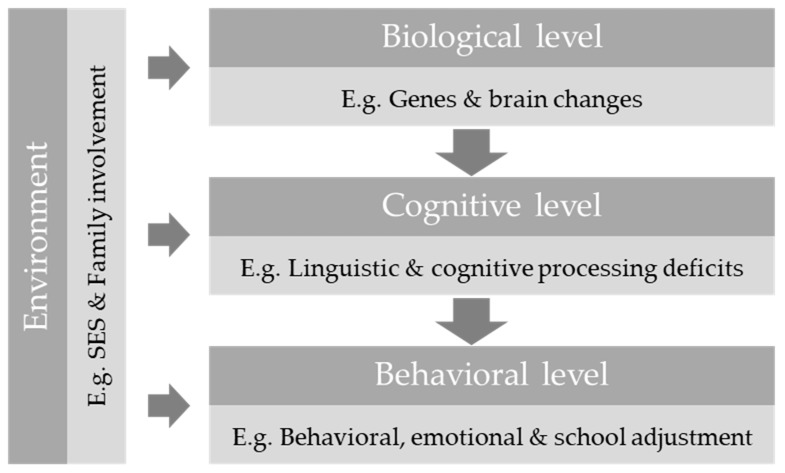
Adaptation of the causal neurodevelopment model of Morton [44].

Altogether, the relation between family involvement and SES, that seemingly affects the quantity or quality of language input, and the behavioral, emotional and school adjustment should be further studied. There are several models available describing the relations between contextual factors and socio-emotional competencies, such as the Positive Youth Development Model [43]. However, we believe that a specific model of neurodevelopment could explain better the relations between these variables in adolescents with DLD, taking into account other important factors in this case such as the linguistic and cognitive variables. In this sense, the Causal Developmental Model of Morton [44] has already been used to compare the causal relations of various developmental disorders, such as dyslexia or autism, and has been beneficial to the training of educational psychologists and to plan educational needs [45]. In particular, Morton [44] proposed a causal model describing three complementary levels of explanation in neurodevelopmental disorders: biological, cognitive, and behavioral, with a fourth environmental level able to influence the other three. Figure 1 depicts the Morton model with an example of several variables that could influence behavioral, emotional and school adjustment in DLD. 

In sum, we cannot ignore that language is a very important tool for the development of social skills and social interaction, and their deficit would result in behavioral, emotional and school adjustment difficulties. Besides, previous studies have shown that deficits in language and social skills can lead to a higher risk of suffering bullying and health problems [21,46]. Nevertheless, most of the reported previous studies on these variables in children and adolescents with DLD have been conducted in Anglo-Saxon contexts in non-inclusive educational settings, and it is unclear whether the results would hold in other languages, educational systems, and cultures. For that reason, our main goal is to explore the relation between DLD and several variables associated to behavioral, scholar and emotional adjustment within a bilingual Spanish-Catalan context, comparing adolescents with DLD with typical development peers. Besides, given that not all adolescents with DLD suffer from behavioral, emotional and social difficulties, we have as a second objective to explore whether environmental variables, such as SES and family involvement, influence language and adaptive behavior and how the former variables might emerge as either risk or protective factors following the model by Morton [44]. 

Thus, and following previous studies, our hypotheses are:

- Adolescents with DLD will present a larger number of behavioral, social and emotional problems, together with worse adaptive skills than their normative peers. This will be reflected in higher scores in the scales of the Behavior Assessment System for Children (BASC) that measure difficulties and lower scores in the scales of the BASC that measure adaptive skills, both self-reported and considering the tutor’s perspective, in contrast to the comparison group.
- With respect to the variables related to the family context, we expected that both SES and family involvement will emerge as protective factors against behavioral, emotional and school adjustment difficulties. Thus, we predicted a negative pattern of correlations between SES/family involvement and the variables measuring difficulties by the BASC and positive correlations between adaptive skills measured by the BASC and familiar variables, for both the self-reported version and the tutor’s version.

## 2. Materials and Methods 

### 2.1. Participants

This longitudinal study was composed of 28 bilingual Spanish-Catalan adolescents. Fourteen participants were adolescents with DLD (six females), and the comparison group included also 14 participants (six females) without any language difficulty. None of the demographic, linguistic and cognitive variables showed gender differences (see Annex I). The participants with DLD were chosen in an incidental way (see Procedure section) from all the schools of Mallorca with children that fulfilled the DLD criteria and were studying the last year of preschool (between 5 and 6 years). For the comparison group, we selected children of the same age and gender who attended the same classroom as the children with DLD, and with as many as possible similarities concerning socioeconomic status (SES) and family language (either Catalan or Spanish). At the end of the follow-up, the participants were between 11 and 12 years old, and were thus at the beginning of adolescence. 

All participants comprising the DLD group were diagnosed with a language delay by the school and the language therapists. In the first phase of our study we confirmed the fulfillment of the DLD criteria, applying different materials when necessary (language and cognitive criteria specifically). Moreover, we checked with the schools’ language therapists for the absence of other incompatible problems such as autism, and with the Balearic Department of Health for the absence of hearing difficulties before forming the groups in the study.

### 2.2. Materials

Language profiles at age 5 were assessed using the standardized test PLON-R: Navarra Oral Language Test-Revised [47]. For language assessment at age 12, we could not use again the PLON-R since its latest application is at six years of age. Because of the absence of specific measures, we used two different measures of language comprehension and language production. Language comprehension was evaluated through the Test of Comprehension of Grammatical Structures (CEG, [48]) and language production was assessed through the sentence repetition subtest of the Developmental Neuropsychological Assessment (NEPSY, [49,50]). These two tests are considered valid indicators of morphosyntax level and they are sensitive for the detection of individuals with DLD [51,52,53,54]. 

Non-verbal IQ was measured at age 5 using the Wechsler Preschool and Primary Scale of Intelligence (WPPSI, [55]). 

To assess behavioral, social and psychological difficulties, we used the Behavior Assessment System for Children (BASC; [56]), specifically its Spanish adaptation [57]. The BASC is a multidimensional assessment tool for children’s and adolescent’s behavior and personality that includes both adaptive and clinical dimensions. We chose the BASC because it also included the variables of interest regarding the school context and it is a tool with adequate psychometric values [58,59]: its test-retest realiability is between *r* = 0.66 and *r* = 0.89, depending on the dimension, meanwhile, its internal consistency reliability is between α = 0.85 and α = 0.96 [57]. We applied two different versions of the BASC, one for the school tutors (T-2 version), and one for the adolescents (S-2 version), both of them appropriate to assess children and adolescents between 6 and 12 years old. The tutor version (T-2) is composed of 149 statements, and each one is answered by means of a four-levels Likert scale (from “never” to “almost always”). Meanwhile, the subject version (S-2) is composed of 146 statements, and each one is answered with a yes/no response. We have used both versions in order to collect two different perspectives regarding the variables in our study, one from the adolescents themselves and the other from an external observer.

Five global dimensions, or composite scales, can be measured by teachers with the BASC T-2: externalization problems (behavioral problems as aggressiveness, hyperactivity, and delinquency; e.g., “He acts without thinking”), internalization problems (depression, anxiety and other similar non-observable difficulties; e.g., “He says: ‘Nobody understands me’), school problems (academic difficulties related to motivation, attention, learning and cognition; e.g., “He makes mistakes because he is not attending”), adaptive skills (prosocial, organizational and other adaptive skills; e.g., “He helps other children”) and a behavioral symptoms index (a global index of behavioral problems; e.g., “He hits other children”). On the other hand, four global dimensions can be self-measured with the BASC S-2: clinical maladjustment (a global index of anxiety due to the different problems experimented by the adolescent, e.g., “I am worried about what other people think about me”), school maladjustment (a global measure of school adaptation; e.g., “I hate the school”), personal adjustment (social and family relationships, self-confidence and self-esteem; e.g., “I think I have good ideas”) and emotional symptoms index (a global index of severe emotional alterations; e.g., “He says: ‘It is all my fault’”).

We also applied a brief questionnaire to evaluate several sociodemographic variables. This questionnaire was created for the present study and was composed of two relevant questions related to SES and family involvement, specifically educational involvement. Both questions were answered by the school tutors. In the case of SES, it was answered by means of a three-level Likert item (“What is the socioeconomic status of the family?”: low, medium and high socioeconomic level). Meanwhile, in the case of family involvement, we used a five-level Likert item (“What is the family involvement in the education process of the child?”: from “none” to “a lot”).

### 2.3. Procedure

The present longitudinal study comprised two phases: an initial identification phase when participants were about 5 years of age (last year of pre-primary school), and a follow-up when participants were 12 years (last course of primary school). 

The research ethics committee (CER; http://www.uib.cat/recerca/estructures/comissions/cer/) of the University of the Balearic Islands approved the study and provided full consent. All parents signed a written informed consent at the beginning of each phase of the study.

A group of trained undergraduate students of advanced academic courses and graduates in Psychology administered all tasks at the participant’s schools. The examiners were also Spanish-Catalan bilinguals and did not know whether the participants belonged to the study group or to the comparison group. Every examiner assessed a participant with DLD and his/her comparison pair in order to minimize differences between examiners.

All identification tests were administered at the beginning of the study when participants were 5 years of age. The BASC, follow-up language tests, and questions related to SES and family involvement were assessed at the end of the follow-up when participants were 12 years of age. In the case of BASC, the examiners were present during its application in order to solve participant’s questions about the items when necessary.

#### 2.3.1. Identification Phase

At first, all schools in Mallorca (Spain) sent us the profiles of all the children with a language delay in the last grade of kindergarten (5 years old) according to their knowledge (*n* = 85). From these 85 profiles, we selected those children (*n* = 23) whose profiles, according to the reports provided by schools’ language therapists, were compatible with the criteria of DLD [3], did not present only an articulatory deficit, did not show autistic traits, and/or had not newly arrived from a non-Catalan speaking community. The language (PLON-R) and non-verbal IQ (WPPSI) of these 23 children were evaluated by our team, and the speech therapists of the schools reported the history of neurological, social, emotional and other difficulties through an open question in a written questionnaire elaborated by us. The interviewer of our team also observed the children in order to confirm they did not show autistic traits. We also asked for their audition records from the Balearic Department of Health. This institution conducts an Otoacoustic Emissions analysis for all children and an audiometric test to those children who fail the previous analysis.

After the assessment, we selected those children who effectively suffered language comprehension and expression problems according to our application of the PLON-R (test cut point for language difficulty when typical score was lower than 25), with an average non-verbal IQ (higher than 85 in our WPPSI application), with no history of cognitive, auditory, social and neurological damage, and without any other disability incompatible with DLD such as the presence of Autism Spectrum Disorder (*n* = 20) [3]. During the follow-up, six participants dropped from the study. For that reason, only 14 children could be included in the current study. 

Every child with DLD was paired by the school with a typically developed child (*n* = 14) of the same age and gender, sharing the same classroom, and with as many as possible similarities concerning socioeconomic status (SES) and family language (either Catalan or Spanish). We recorded the same variables for both the DLD and the comparison group. None of the demographic variables showed differences between groups at this phase, while all the linguistic proficiency measures displayed significant differences (see Table 1 and Table A1). 

#### 2.3.2. End of Follow-Up

At the end of the follow-up when participants were about 12 years, language, behavior, emotional, socioeconomic and family aspects were evaluated. Language comprehension and production was assessed through the CEG [48] and the sentence repetition subtest of the NEPSY [49,50]. Results revealed significant differences at 12 years between both groups in language comprehension and production (see Table 1). 

The BASC and the questions related to SES and family involvement were also assessed at this phase. Participants’ tutors specified which SES category the families of the children fit into. To this end, they responded to a three–choice question with the following SES categories: low, medium, and high.

Moreover, we consulted the schools’ language therapists in order to gather several variables that helped us to better understand what could have occurred in the gap between the two phases of our study. For that reason, we asked about the presence of school or external support and special educational needs or curricular adaptations during the primary education for the adolescents in our sample. In this regard, only four participants had special educational needs and curricular adaptations according to the school data, and all of them were in the DLD group. Referring to external support, only four adolescents in the DLD group had it; three took particular classes outside the school and one received speech and language therapy. None of the participants received psychological intervention. 

### 2.4. Design and Data Analyses

In order to test our first hypothesis regarding school adaptation and behavioral and emotional problems between both groups, we conducted diverse Mann-Whitney tests (*U)*. We used non-parametric analyses due to the limited sample size and the fact that not all assumptions for the parametric way were fulfilled, such as data normality. For the BASC teacher rating scales (T2), Group (DLD, Comparison) was considered as the independent variable, while Externalization Problems, Internalization Problems, School Problems, Adaptive Skills, and the Behavioral Symptoms Index were the dependent variables. For the BASC subject rating scales (S2), Group (DLD, Comparison) acted as the independent variable too, with Clinical Maladjustment, School Maladjustment, Personal Adjustment and Emotional Symptoms Index acting as dependent variables. We also calculated the effect sizes with the non-parametric Cliff’s Delta (δ), which was interpreted following this estimator’s categories [60]: small effect (δ = 0.2), medium effect (δ = 0.3) and large effect (δ = 0.5). SPSS v25 statistical software was used for all the non-parametric statistical analyses. A significance level of *p* < 0.05 was used for all comparisons.

Additionally, non-parametric correlations and a partial least square (PLS) structural equation model between Family Involvement, SES, and the aforementioned dependent variables were conducted in order to test our second hypothesis, which referred to the putative influential role of SES and Family Involvement on linguistic, cognitive and adaptive variables. For the correlation sets, the sample was used both as a whole and also split into two groups, but for the path analysis, the sample was used only as a whole because of the small sample size. Therefore, the relations between the key indicators of this work were further examined using a PLS structural equation model following the model by Morton [44]. The PLS model estimates the associations between the constructs by means of ordinary least regressions, allowing a better understanding of the mediating role of the different variables [61]. Moreover, some of the advantages of using PLS are their low sample size demands and the flexibility of their distributional assumptions burdens [61,62].

Regarding the evaluation of the PLS model, the proceeding assesses two distinct parts: the measurement models and the structural model, sequentially. The measurement models should be assessed first, including their indicator loadings (values > 0.7 are adequate), reliability, and validity. As for the two latter, the Cronbach alpha (values between 0.6 and 0.7 are satisfactory) and the average variance extracted (AVE; values > 0.5 are acceptable), respectively, are of common use [63]. If the measurement model evaluation is adequate, the structural model results can be examined, including possible collinearity issues, the coefficient of determination (*R*^2^), and the path coefficients [61]. The variance inflation factor (VIF; values < 3 are acceptable) assesses the collinearity of the structural model. The *R*^2^ reflects the explained variance in each of the endogenous variables, and its values range from 0 (no explanatory power) to 1 (highest explanatory power). As a guideline [63], explanatory power can be considered as weak, moderate and substantial according to the following values: 0.25, 0.5 and 0.75, respectively. Finally, the path coefficients can be used to interpret the PLS model results in terms of the significance and strength of the relations in the structural model, that is, how the variables interact with each other. In this sense, this statistic can be understood as a standardized regression coefficient (β), which usually ranges from -1 to 1, indicating a negative or positive relation, respectively, if significant. SmartPLS (v.3.2.8) [64] was used to analyze the data. 

## 3. Results

### 3.1. Behavioral, Emotional and School Adaptive Variables

We analyzed the differences between both groups in the composite scales that configure the dimensions of the BASC for both versions. 

**Figure 2:**
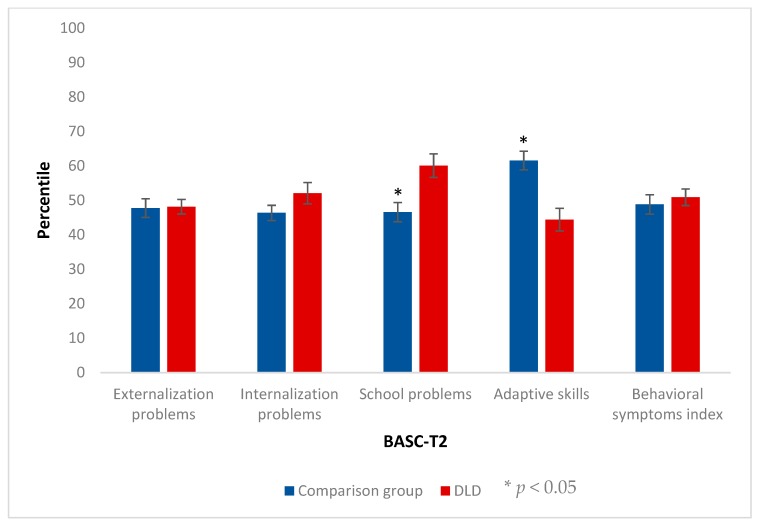
Results for the composite scales of the Behavior Assessment System for Children (BASC) teacher rating scales (T2). The bars represent the means and the error bars represent the standard error.

Regarding the tutors’ version (T2), we found statistically significant differences in two of the dimensions assessed (see Figure 2). More specifically, the analysis revealed large effect sizes in School Problems (*U* = 39, *p* = 0.012, δ = 0.57) and Adaptive Skills (*U* = 28, *p* = 0.002, δ = 0.69). On the contrary, Externalization Problems (*U* = 85, *p* = 0.769, δ = 0.07), Internalization Problems (*U* = 71.5, *p* = 0.343, δ = 0.21) and Behavioral Symptoms (*U* = 49.5, *p* = 0.119, δ = 0.36) did not show significant differences between groups.

**Figure 3:**
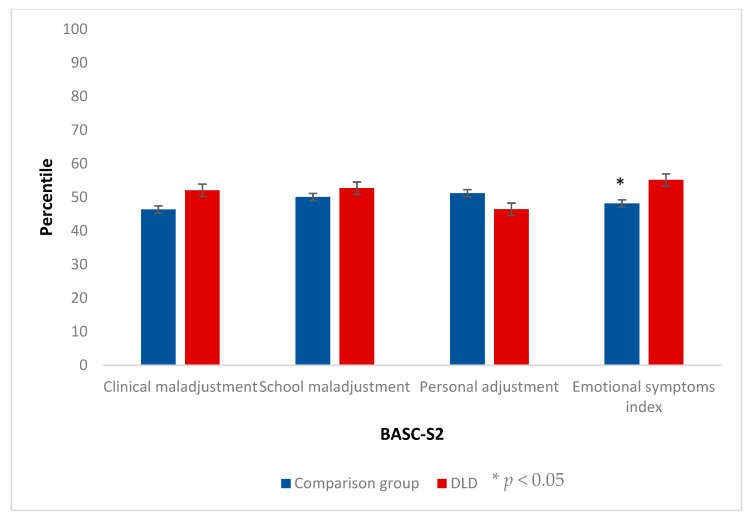
Results for the composite scales of the BASC participant rating scales (S2). The bars represent the means and the error bars represent the standard errors.

Regarding the participant’s version (S2), only the Emotional Symptoms Index (*U* = 53.5, *p* = 0.041, δ = 0.45) reached significance, with a medium effect size (see Figure 3). The Clinical Maladjustment (*U* = 63.5, *p* = 0.112, δ = 0.35), the School Maladjustment (*U* = 82, *p* = 0.451, δ = 0.16), and the Personal Adjustment (*U* = 66, *p* = 0.140, δ = 0.33) scales did not reveal significant differences between both groups. 

### 3.2. Relation between SES and Family Involvement with Behavioral, Emotional and Adaptive Skills

Table 2 displays the correlations between SES, Family Involvement and the BASC variables (for both versions, tutors, and participants). 

Contrary to our hypothesis, we found no significant correlation between SES and the variables included in the BASC. This lack of significant correlations might be due to the control applied to this variable in our samples since we sought normative participants who were comparable to DLD participants in terms of sociodemographic data. However, these results hold when both groups were considered separately, and none of the variables of the BASC correlated significantly with SES.

Nevertheless, as expected, Family Involvement correlated with many of the BASC variables when both groups were taken together (see Table 2). We found significant negative correlations between Family Involvement and Internalization Problems, School Problems, Behavioral Symptoms Index and the Emotional Symptoms Index. Meanwhile, we also found significant positive correlations between Family Involvement, Adaptive Skills, and Personal Adjustment.

Considering the Comparison group and the DLD group separately, only Family Involvement correlated negatively with School Problems in both groups. Differently, the Behavioral Symptoms Index correlated negatively with Family Involvement only in the DLD group, while Personal Adjustment informed by participants correlated negatively with Family Involvement only in the Comparison group. Nevertheless, given that correlation analyses split by group include only half of the sample, its power decreases substantially. Thus, these results must be interpreted with caution.

Concerning the PLS path model estimation, results regarding the measurement model can be found in Table 3. 

**Figure 4:**
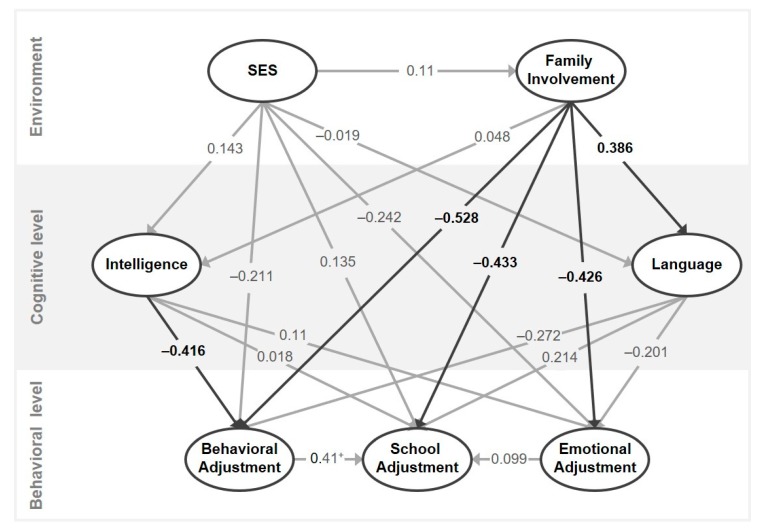
Model displaying the relations between the variables included in the model, which parallels the one by Morton (2004). Numbers represent path coefficients (β), and both paths and numbers in bold reflect significant effects among constructs. ^+^ indicates a marginal effect.

As can be seen, the only latent variable suitable for assessment was Language (the rest of the indicators were single-item variables, thus, they have values of 1 in their indicator loadings, alpha and AVE, and therefore are satisfactory). As for Language, both the Sentence Repetition (SR) test of the NEPSY and the PLON-R measures showed acceptable and significant indicator loadings. However, while the Test of Comprehension of Grammatical Structures (CEG) loading did not reach significance we kept it in the latent variable since it was close to the threshold value and both the Cronbach’s alpha and the AVE were adequate [65]. Regarding the structural model results, no variable showed collinearity, and the target *R*^2^ results of the behavioral variables revealed moderate explained variance (see Table 3). Moreover, the path coefficients estimate of the structural model showed that Family Involvement had a negative influence on all behavioral variables: Behavior Adjustment: β = ‒0.528, *p* < 0.0001; School Adjustment: β = ‒0.433, *p* = 0.026; Emotional Adjustment: β = ‒0.426, *p* = 0.019 (see Figure 4). Intelligence did reveal a negative association with School Adjustment (β = ‒0.416, *p* = 0.022). However, note that the scale of the BASC items (comprising the behavioral level section of the model) becomes larger as a function of worse adjustment, and, therefore, in this context a greater Family Involvement and Intelligence imply a better adjustment. Furthermore, Family Involvement did have an effect on the Language latent variable (β = 0.386, *p* = 0.012). Additionally, Behavioral Adjustment showed a marginal effect on School Adjustment (β = 0.41, *p* = 0.066). No other direct (*p*s > 0.202) or indirect effects (*p*s > 0.133) reached significance, and thus no other influence was observed in the model for the other variables.

## 4. Discussion

The present study aimed at exploring the behavioral, emotional and scholar adjustment in adolescents with DLD in contrast to a comparison group, and to explore whether SES and family involvement would be confirmed as protective factors against adaptive difficulties in adolescents with a language disorder. Although the small sample size hampers the generalization of the study, our findings suggest that DLD is not an exclusively language-focused difficulty.

Results partially confirmed our first hypothesis, since we found that adolescents with DLD have more school problems and less adaptive skills than their normative peers. Other studies have found similar results [4,6,19] showing that children and adolescents with DLD possess less adaptive abilities such as social skills, leadership, and adaptive skills. More school difficulties in children and adolescents with DLD have also been reported in many studies [66,67], not only in an academic sense but also in the adjustment to the scholar context [4,68]. Contrary to other studies that documented more internalized behaviors, such as depression and anxiety [4], and/or externalized ones, such as aggression and hyperactivity [68] in individuals with DLD, our results do not permit to corroborate the conclusions of other similar studies. Furthermore, the results of the present study in terms of emotional disturbances, such as depression and anxiety (internalized problems), have to be taken with caution given the sample size of the present study.

With respect to the internalized problems, previous works have found a higher percentage of internalized problems in children with dyslexia (a comorbid disorder with DLD), but there is also a great heterogeneity between studies. Accordingly, studies with samples recruited in clinical settings have greater possibilities to report internalized difficulties than samples recruited in scholar settings [69], and higher levels of internalized problems are found with more probability when children grow [70]. In consonance with these results, Font-Jordà et al. [4] also found higher internalized symptoms in Spanish-Catalan children with DLD who were recruited in clinical settings, contrary to our study in which the sample was recruited in a school setting. Furthermore, our sample is composed of adolescents still at primary school. Thus, differences in recruitment settings and the age of the samples between studies could explain the lack of internalized problems in the current sample. 

With respect to the externalized problems, Font-Jordà et al. [4] did not report more externalized behaviors in a clinical sample of Spanish-Catalan children with DLD. In contrast, previous studies have shown that externalized behaviors increase from kindergarten to grade four in children with a low language level [11,12,23]. Nevertheless, this relation is greater for males and is mediated by other variables such as peer rejection [68]. Therefore, externalized behaviors seem not to be a group characteristic of individuals with DLD and they are mediated by other variables (such as gender and peer rejection).

It is worthy of note that, in our study, the higher adaptive difficulties and school problems found in adolescents with DLD were appreciated only by the adolescent’s tutors. Meanwhile, participants in both groups did not differ when assessing themselves in the BASC, except for larger emotional symptoms index in the DLD group. A similar pattern of results has been found in other comorbid difficulties with DLD, such as dyslexia [69], where individuals display more difficulties when the informant was the teacher versus themselves. Besides, tutors and adolescents have different perceptions regarding their problems. On the one hand, tutors mainly focus on attentional and learning problems (school problems) and adaptive skills (leadership and social skills), which they perceive with relative ease and can relate to class functioning and daily life. On the other hand, adolescents with DLD seem to report more emotional symptoms than their peers. Maybe this difficulty is better perceived by the participants themselves because it is an internal problem which is harder to be noticed by other people, like the tutors in this case. Another possibility is that the amount of personal distress perceived by adolescents with DLD was high enough to yield significant differences, even when the self-reported measures might be less prone to reveal difficulties in this particular group. Therefore, in line with other works, the results of this study report that adolescents with DLD are not conscious of their lower level of social skills, although they note their emotional suffering [25].

Taking these results together, one might argue that tutors and adolescents are not fully aware of the problems reported by each other. This is an important finding because it highlights areas in which it is necessary to work with individuals with DLD. If they are experiencing more emotional symptoms than their peers and show more school problems and less adaptive skills, they could be at more risk of exclusion or rejection without having the optimal coping strategies [19,68]. 

With regard to our second hypothesis involving the protective factors, we expected both higher SES and family involvement to be associated with lower behavioral, emotional and school adjustment disturbances. The present study revealed that only larger family involvement was related to a lower array of difficulties, as measured with the BASC. The lack of relation of the BASC variables with SES could be explained by the control made on the SES variable, with no significant departing differences between DLD and comparison groups. Nevertheless, this lack of significance is maintained when analyses are performed separately for each group. Probably the socioeconomic situation of Spain (Position 34 of the international socioeconomic ranking and good quality of live [71]) and its compensating measures for poverty might be helping to equalize some of the socioeconomic consequences in adolescents with DLD in our country, contrary to results found in other countries such as Mexico (Position 71 of the international socioeconomic ranking and a low standard of living [29,71]).

Meanwhile, family involvement is related to many of the variables as hypothesised, since it correlated positively with adaptive skills and personal adjustment, being both useful resources against social, emotional and adaptative difficulties capable of disrupting adolescent’s well-being. Moreover, family involvement was negatively correlated with internalization problems, school problems, and behavioral and emotional symptoms. Thus, family involvement could be an important protective factor for individuals with DLD as it has already been considered as a predictor of social performance and behavioral difficulties in typically developing persons [41]. However, it is noteworthy to mention that the opposite pattern can also be possible, and a poor involvement or an inattentive family could make the child’s social development not optimal. In this sense, Lisa and colleagues [72] found that those children with DLD who had mothers with a high degree of maternal stress at home were more likely to be bullied at school. This is explained by the fact that these children had more trouble acquiring adequate social competencies. Moreover, a limitation of our study in this field is the non-use of a validated measure of family involvement, such as Family Involvement Questionnaire Elementary (FIQ-E) [36] or any similar tool. This aspect ought to be taken into consideration in further studies.

This outcome was further corroborated by the PLS model and highlights the direct influence of an environmental factor, such as family involvement, as a protective factor against behavior, school, and emotional adaptive difficulties. It is worthy of note that family involvement and SES were not related, and thus SES did not mediate the influences of family involvement on any of the reported variables of adjustment. Our results can be interpreted with the Developmental Causal Model of Morton [42], in which the environmental domain could affect the cognitive and behavioral level. In this sense, family involvement also positively influenced language skills at the latent level, in line with previous studies that have used a correlational approach [20,28]. However, we found no mediating influences of intelligence or language (cognitive level) on outcome variables related to low adjustment (behavioral level). Other studies have also found that family variables such as maternal mental health could influence social, emotional and behavioral functioning in children with DLD [20,28]. Besides, previous studies have found more insecure patterns of attachment in children with DLD [38]. Therefore, it is important to encourage the families to get involved in the education of children and adolescents and to take families into account in the programs and protocols designed to aid individuals with DLD.

In conclusion, our findings highlight the importance of some environmental variables, such as family agents, when considering the well-being of adolescents with and without DLD. In the case of persons with DLD, it is crucial to stimulate language skills as these configure their main handicap, but it is equally important to tackle other issues that fall within the same global problem, such as the fluid and meaningful communication in their daily life, their social and leadership skills, the relationship with their parents and their school, and their emotional competence. An integral effort that takes into account all these aspects is necessary and might help to prevent and protect against other related difficulties that could appear when the aforementioned skills are not optimal, such as bullying and victimization. 

The findings of this study must be interpreted with caution mainly because of the relatively small sample size that hampers the generalization of the study. Further studies might want to complement and enrich the present results by contrasting the perceptions of parents as well, as the BASC also allows researchers to collect the corresponding data in this regard.

## 5. Conclusions

Adolescents with DLD not only present language problems, but they also experience several difficulties related to behavior, emotional and school adjustment and well-being. Our study shows that adolescents with DLD have more school problems and less adaptive skills than their normative peers, and suffer more in the emotional domain. Furthermore, family involvement arises as an important environmental factor that influences both the language level and behavioral adjustment in adolescents with and without DLD and can be considered an important protective factor of mental health. For this reason, an integral approach to well-being in DLD, but also in normative adolescents, should consider not only the individuals, but also schools, families, and their social and emotional resources.

## Figures and Tables

**Table 1:** Demographic and linguistic data of participants.

		Comparison Group	DLD	Statistic and *p*
		Mean (SD)	Mean (SD)	
*n*		14	14	
Age at identification		5.85 (0.25)	5.74 (0.24)	*U* = 68.5, *p* = 0.171
Age at follow-up		11.85 (0.25)	11.74 (0.24)	*U* = 66.5, *p* = 0.143
SES		2 (0.47)	1.85 (0.55)	*U* = 56, *p* = 0.468
Family involvement		4.14 (0.83)	2.93 (1.27)	*U* = 44, *p* = 0.011
**Gender**				
Male		8	8	X^2^ = 0.00, *p* = 1.00
Female		6	6	
**Language used at school**				
Catalan		8	10	X^2^ = 2.22, *p* = 0.329
Spanish		2	3	
Bilingual		4	1	
**Family language**				
Catalan		6	3	X^2^ = 1.53, *p* = 0.465
Spanish		7	10	
Bilingual		1	1	
**Identification phase at 5 years of age**				
Nonverbal-IQ (WPPSI)		110 (12.2)	102.1 (9.9)	*U* = 63.5, *p* = 0.112.
Typical score language (PLON-R)		55.9 (22.6)	19.8 (6.7)	*U* = 0.00, *p* < 0.000
**Language at the end of follow-up (12 years)**				
Percentile language production (SR NEPSY)		60.7 (16.4)	29.4 (22.4)	*U* = 30.5, *p* = 0.002
Percentile language comprehension (CEG)		64.3 (24.0)	40.7 (24.5)	*U* = 52.0, *p* = 0.033

SES = socioeconomic status; PLON-R = Navarra Oral Language Test-Revised, WPPSI = Wechsler Preschool and Primary Scale of Intelligence; SR = Sentence repetition; NEPSY = Developmental Neuropsychological Assessment; CEG = Test of comprehension of grammatical structures.

**Table 2:** Spearman correlation coefficients (ρ) between socioeconomic status (SES), family involvement and the variables of the BASC.

	Both Groups		Comparison Group		DLD Group	
	SES	Family Involvement	SES	Family Involvement	SES	Family Involvement
**Tutor form**						
Externalization problems	-0.22	−0.05	−0.47	−0.005	−0.01	−0.16
Internalization problems	−0.20	−0.36 *	−0.31	−0.36	−0.15	−0.54
School problems	−0.02	−0.58 **	0.23	−0.58*	−0.24	−0.69 **
Adaptive skills	0.18	0.42 **	−0.23	0.37	0.44	0.31
BSI	−0.24	−0.46 *	−0.18	−0.46	−0.23	−0.61 *
**Subject form**						
Clinical maladjustment	-0.01	−0.18	0.23	−0.18	−0.36	−0.16
School maladjustment	-0.01	−0.04	0.60	−0.17	−0.30	0.11
Personal adjustment	0.21	0.34 *	0.00	0.65 *	0.53	0.36
ESI	−0.16	−0.36 *	−0.08	−0.42	−0.41	−0.45

SES = socioeconomic status; BSI = Behavioral symptoms index; ESI = Emotional symptoms index. ** *p* < 0.01; * *p* < 0.05.

**Table 3:** Measurement and structural model statistics of the study variables.

	Measurement Model				Structural Model	
	Indicator Loads					
	Load	*p*	A	AVE	VIF	*R* ^2^
**Environment level**						
SES	1	<0.001	1	1	1	
Family Involvement	1	<0.001	1	1	1	0.012
**Cognitive level**						
Intelligence (WPPSI)	1	<0.001	1	1	1	0.024
Language			0.72	0.63		0.148
Grammar comprehension (CEG)	0.682	0.205			1.34	
Sentence production (SR, NEPSY)	0.869	<0.001			1.43	
PLON-R	0.822	0.002			1.49	
**Behavioral level**						
Behavioral adjustment	1	<0.001	1	1	1	0.428
School adjustment	1	<0.001	1	1	1	0.703
Emotional adjustment	1	<0.001	1	1	1	0.348

*Note*. A = Cronbach’s alpha, AVE = average variance extracted, VIF = variance inflation factor, *R*^2^ = coefficient of determination, SES = socioeconomic status, Intelligence = Preschool and Primary Scale of Intelligence, NEPSY = Developmental Neuropsychological Assessment, SR = Sentence Repetition, CEG = Comprehension of Grammatical Structures, PLON-R = Navarra Oral Language Test-Revised, Behavioral adjustment: Behavioral Symptoms Index (BSI, BASC-T2), School adjustment = School problems (BASC-T2), Emotional adjustment = Emotional Symptoms Index (ESI, BASC-S2).

## Appendix A

**Table A1:** Sociodemographic data split by gender.

	Female		Male		Statistic	
	Comparison Mean(SD)	DLD Mean(SD)	Comparison Mean(SD)	DLD Mean(SD)	Comparison Male-Female	DLD Male-Female
*n*	6	6	8	8		
Age at identification	5.87 (0.27)	5.63 (0.17)	5.82 (0.25)	5.82 (0.25)	*U* = 20	*U* = 13.5
Age at follow-up	11.88 (0.27)	11.63 (0.18)	11.84 (0.25)	11.81 (0.26)	*U* = 20	*U* = 14.5
SES	2 (0.0)	2 (0.63)	2 (0.7)	1.71 (0.49)	*U* = 12.5	*U* = 16
Family involvement	4.5 (0.55)	2.5 (1.22)	3.87 (0.99)	3.25 (1.28)	*U* = 15	*U* = 15.5
**Language used at school**						
Catalan	4	5	4	5		
Spanish	0	0	2	3	X^2^ = 1.75	X^2^ = 1.48
Bilingual	2	1	2	0		
**Family language**						
Catalan	4	2	2	1		
Spanish	1	4	6	6	X^2^ = 5.05	X^2^ = 3.79
Bilingual	1	0	0	1		
**Identification phase at 5 years of age**						
Nonverbal-IQ (WPPSI)	115.5 (11.02)	106.83 (10.74)	105.87 (12.11)	98.62 (8.38)	*U* = 12	*U* = 12.5
Typical score language (PLON-R)	59.67 (20.48)	19.67 (9.69)	53.12 (25.08)	20.0 (4.14)	*U* = 17	*U* = 19.5
**Language at the end of follow-up (12 years)**						
Percentile language production (SR NEPSY)	58.33 (17.22)	21.83 (20.2)	62.5 (16.69)	35.12 (23.7)	*U* = 19	*U* = 16.5
Percentile language comprehension (CEG)	70.83 (24.98)	41.67 (30.6)	59.37 (23.67)	40.0 (21.21)	*U* = 16.5	*U* = 23

SES = Socioeconomic status; PLON-R = Navarra Oral Language Test-Revised, WPPSI = Wechsler Preschool and Primary Scale of Intelligence; SR = Sentence repetition; NEPSY = Developmental Neuropsychological Assessment; CEG = Test of comprehension of grammatical structures.
